# Mice lacking mitochondrial ferritin are more sensitive to doxorubicin-mediated cardiotoxicity

**DOI:** 10.1007/s00109-014-1147-0

**Published:** 2014-04-13

**Authors:** Federica Maccarinelli, Elena Gammella, Michela Asperti, Maria Regoni, Giorgio Biasiotto, Emilia Turco, Fiorella Altruda, Silvia Lonardi, Laura Cornaghi, Elena Donetti, Stefania Recalcati, Maura Poli, Dario Finazzi, Paolo Arosio, Gaetano Cairo

**Affiliations:** 1 Department of Molecular and Translational Medicine, University of Brescia, Viale Europa 11, 25123 Brescia, Italy; 2 Department of Biomedical Sciences for Health, University of Milano, Via Mangiagalli 31, Milan, Italy; 3 Department of Molecular Biotechnology and Health Sciences, University of Torino, Via Nizza, 10126 Torino, Italy

**Keywords:** Ferritin, Oxidative damage, Mitochondria, Doxorubicin

## Abstract

**Abstract:**

Mitochondrial ferritin is a functional ferritin that localizes in the mitochondria. It is expressed in the testis, heart, brain, and cells with active respiratory activity. Its overexpression in cultured cells protected against oxidative damage and reduced cytosolic iron availability. However, no overt phenotype was described in mice with inactivation of the FtMt gene. Here, we used the doxorubicin model of cardiac injury in a novel strain of FtMt-null mice to investigate the antioxidant role of FtMt. These mice did not show any evident phenotype, but after acute treatment to doxorubicin, they showed enhanced mortality and altered heart morphology with fibril disorganization and severe mitochondrial damage. Signs of mitochondrial damage were present also in mock-treated FtMt^−/−^ mice. The hearts of saline- and doxorubicin-treated FtMt^−/−^ mice had higher thiobarbituric acid reactive substance levels, heme oxygenase 1 expression, and protein oxidation, but did not differ from FtMt^+/+^ in the cardiac damage marker B-type natriuretic peptide (BNP), ATP levels, and apoptosis. However, the autophagy marker LC3 was activated. The results show that the absence of FtMt, which is highly expressed in the heart, increases the sensitivity of heart mitochondria to the toxicity of doxorubicin. This study represents the first in vivo evidence of the antioxidant role of FtMt.

**Key message:**

- Mitochondrial ferritin (FtMt) expressed in the heart has a protective antioxidant role.
- Acute treatment with doxorubicin caused the death of all FtMt^−/−^ and only of 60 % FtMt^+/+^ mice.
- The hearts of FtMt^−/−^ mice showed fibril disorganization and mitochondrial damage.
- Markers of oxidative damage and autophagy were increased in FtMt^−/−^ hearts.
- This is the first in vivo evidence of the antioxidant role of FtMt.

**Electronic supplementary material:**

The online version of this article (doi:10.1007/s00109-014-1147-0) contains supplementary material, which is available to authorized users.

## Introduction

Mitochondrial ferritin (FtMt) is a recently identified ferritin type that accumulates specifically in the mitochondria [1]. Human FtMt, which is encoded by an intronless gene, is synthesized as a precursor with a long N-terminal targeting sequence that is cleaved in the mature protein. The mature FtMt has a functional ferroxidase center and forms stable ferritin shells that readily accumulate iron [2]. Its 3D structure is analogous to that of the H-ferritin [3]. The FtMt transcript does not contain a functional iron responsive element (IRE) sequence, and thus, FtMt expression is not controlled by intracellular iron levels. In humans, FtMt was found to be expressed in the testis [4], neurons [5], and in the erythroblasts of subjects with genetic or acquired sideroblastic anemia [6], where it is responsible of the mitochondrial iron deposits of the sideroblasts. Since human FtMt is not detectable in cultured cells, most of the data on its function have been obtained by analyzing the phenotype of overexpressing cells. These studies showed that, by virtue of its ferroxidase activity, FtMt actively sequesters iron inside the mitochondria at the expense of cytosolic iron [7]. It also reduces iron-mediated oxidative damage of mitochondria [8] and rescues some defects caused by frataxin deficiency in yeast [9] and HeLa cells [10]. More recently, it has been shown that overexpression of FtMt in K562 erythroid cells reduced Jak/STAT signaling and increased apoptosis [11]. In sideroblasts, erythroid progenitors FtMt expression occurred at the early stage of cell differentiation and was accompanied by reduced iron availability and increased apoptosis [12, 13]. Moreover, FtMt expressing cells transplanted in nude mice grew more slowly than their control counterpart [14]. Altogether, FtMt seems to protect mitochondria against iron-dependent oxidative damage and also modifies cellular iron distribution by attracting iron from the cytosol to mitochondria [15]. Specific antibodies have been raised against mouse FtMt that were used to study its distribution in mouse organs. It was found that FtMt is strongly expressed in the testis, particularly in the spermatocytes, and also in the heart, kidney, Purkinje cells, and some neurons, and generally in cells with high respiratory activity that actively use iron enzymes and produce reactive oxygen species (ROS) [16]. In line with the results obtained in the cell lines, FtMt was also found to decrease the sensitivity of mitochondria to oxidative damage in SLA mice [17] and to protect neuronal cells from oxidative damage [18–20]. The role of FtMt in neurodegenerative diseases has been recently reviewed [21]. However, the recently described FtMt-deficient mice in the C57BL/6J strain did not show any evident phenotype and produced siderocytes/sideroblasts similar to the control FtMt^+/+^ even under conditions of vitamin B6 deficiency [22]. It is unlikely that the lack of phenotype in FtMt deficiency is due to redundancy of the function, since FtMt is the only known mitochondrial iron storage protein. Although FtMt appears dispensable under physiological conditions, it may protect against damages under specific conditions. FtMt is highly expressed in the heart; thus, we considered that it may protect its mitochondria against oxidative damage, in particular from the injury induced by doxorubicin (Dox), a well-characterized anthracycline whose extensive use for the cure of a variety of tumors is hampered by a recognized cardiotoxicity [23–25]. Anthracyclines, including Dox, possess a high affinity for cardiolipin, a negatively charged phospholipid of the inner mitochondrial membrane [26], and hence, they are retained at high concentrations in the mitochondrial compartment. Confocal microscopy experiments showed that in H9c2 cardiomyocytes, Dox localized to mitochondrial sites of redox cycling and ROS formation [27]. Therefore, mitochondria have been repeatedly suggested as the most important target for anthracyclines cardiotoxicity [24]. In this context, the availability of intracellular reactive iron, which catalyzes ROS formation, appears to be determinant for Dox cardiotoxicity, as shown by the protective effect of iron chelation in patients and in experimental models [28], whereas iron overload have been shown to exacerbate the cardiotoxic effects of the drug [29–32]. In particular, mitochondrial iron may play a significant role in leading to elevated mitochondrial ROS formation, and it has been recently shown that overexpression of FtMt can protect HeLa cells from Dox toxicity [33] and that Dox treatment strongly induces FtMt expression in neonatal rat cardiomyocytes [34]. Moreover, a recent study showed that Dox cardiotoxicity is mediated by mitochondrial iron accumulation and that heart-specific deletion of mitochondrial iron exporter ABCB8 increased mice sensitivity to Dox cardiotoxicity, whereas its overexpression was protective [34].

In this study, we tested the hypothesis that the heart of FtMt-deficient animals might be more sensitive to cardiotoxic drugs; with this aim, we used a model of acute Dox cardiotoxicity in a novel strain of FtMt-null mice to evaluate the role of FtMt in heart protection from anthracycline-dependent oxidative injury.

## Materials and methods

### Animals

All the procedures followed animal protection laws and institutional guidelines of the European Convention for the Protection of Laboratory Animals. The study was approved by the Institutional Animal Care and Use Committee of the University of Brescia and the Italian Ministry of Science and Research.

### Gene targeting construct

A 5,030-bp cassette that included the Lac-Z gene followed by the hybrid pGK-EM7 promoter and the Neo gene was cloned into the pBluescript-SK (pBSK) plasmid. The cassette was flanked by a 5′ homology arm of 275 bp upstream the FtMt start codon and a short 3′ arm of 270 bp downstream the FtMt stop codon. The construct named pBSK + 5-b-n-3 was verified by DNA sequencing and then used together with the vector pBSK + moMtF of 21.5 kbp containing the full mouse gene and flanking sequences in the recombineering system [35] to obtain the gene targeting vector that had homology arms at 5′ and 3′ of about 8 kbp. The construct was then used for electroporation of mouse ES cells, which were then selected in G418 medium, and one clone was microinjected in embryos. The born mice showed about 50 % chimerism and were crossed with C57BL/6J mice, and the newborn genotyped by PCR as described below. Mice were bred in mixed C57BL/6J x 129 genetic background.

### Genotyping

The mice were genotyped by PCR using a common Fwd-primer upstream the 5′-arm and two different Rev-primers, one specific for the mouse mitochondrial ferritin (moFtMt) gene (see Table 1), using the following PCR conditions: 5 min at 95 °C, 5 cycles (30 s at 94 °C, 30 s at 50 °C, 45 s at 72 °C), 30 cycles (30 s at 94 °C, 30 s at 55 °C, 45 s at 72 °C), followed by 10 min at 72 °C.

**Table 1:** Primers and Taqman assays used

Genotyping primers	
B5-Fwd	5′-ACGCGTCGACCTTGTGTTAGTAATTCAGCC-3′
mT4-Rev	5′-CAGAGTATGTAAGTCCAGCAGC-3′
LacZ-Rev	5′-GGGACGACGACAGTATCGGCCT-3′
RT-PCR primers	
BGAL-FwdNew	5′-GCACGGTTACGATGCGCCCA-3′
BGAL-RevnNew	5′-GCGCTGGAGTGACGGCAGTT -3′
mT1-For	5′-TATTTCCTTCGCCAGTCCCTG-3′
mT4R-Rev	5′-CAGAGTATGTAAGTCCAGCAGC-3′
mHPRT1-For	5′-GCTTGCTGGTGAAAAGGACCTCTCGAAG-3′
mHPRT1-Rev	5′-CCCTGAAGTACTCATTATAGTCAAGGGCAT-3′
*TaqMan gene*	*Assay ID*
HO-1	Mm00516005_m1
BNP	Mm01255770_g1
p53	Mm00519571_m1
Rn18s	Mm03928990_g1

The genotyping primers were used to verify the presence of FtMt gene or of LacZ gene in the FtMt locus. RT-PCR primers were used for identification of the transcripts of FtMt, β-Gal, and HPRT1 in the testis of the mice. The TaqMan gene expression assays were used to quantify HO-1, BNP, p53, and Rn18s transcripts in the heart of the treated mice

### DOX treatments

To induce cardiotoxicity in the survival studies, 84-day-old female mice were injected intraperitoneally with saline or a single dose (15 mg/kg of body weight) of Dox (Sigma, Milan, Italy) and then, the mice were followed for 30 days. In other experiments, female mice were treated as above and sacrificed after 4 days for the analysis of the heart, or male mice were sacrificed after 30 days for analysis of the testis. The organs were harvested, weighted, frozen in liquid nitrogen, and stored at −80 °C.

### Quantitative real-time polymerase chain reaction (qRT-PCR)

Total heart or testis RNA purified using TRI reagent® (Sigma) was reverse transcribed into cDNA with Proto Script M-MuLV First Strand cDNA Synthesis Kit (New England Biolabs, Italy), and the obtained cDNA served as a template for real-time PCR, based on the TaqMan methodology (Life Technologies). Primers (Applied Biosystems) and parameters are described in detail in Table 1. For evaluation of beta-galactosidase (β-gal) and FtMt transcripts, we used RT-PCR with the primers described in Table 1 and cycling conditions as follows: 5 min at 95 °C, 30 cycles (30 s at 94 °C, 30 s at 60 °C, and 30 s at 72 °C), followed by 10 min at 72 °C.

### Western blotting

Tissue lysates were prepared in RIPA buffer, incubated on ice for 30 min, and centrifuged at 13,000 rpm for 10 min. Proteins in supernatant were separated on non-denaturing or SDS-PAGE and transferred onto nitrocellulose membranes (GE Healthcare, Milan, Italy). Membranes were processed and incubated with primary antibodies against mouse FtMt, H and L ferritins [16], β-gal, LC3 and GAPDH (Sigma), and horseradish peroxidase (HRP)-conjugated secondary antibodies. The antigens were detected using an immunodetection kit (ECL Basic, Amersham Biosciences). For evaluation of ferritin iron, the non-denaturing gels were stained with Prussian blue and then the color enhanced using diaminobenzidine (DAB) and H_2_O_2_ (Ft-iron). For visualization and densitometry, we used the Kodak Image Station 440CF (Kodak).

### Determination of ATP content

Heart tissue samples (10 mg) were homogenized in perchloric acid, and ATP was measured by a colorimetric assay using a commercial kit (ATP Colorimetric assay kit; Biovision, Italy) following manufacturer’s instructions.

### Immunohistochemistry

Mouse testes were dissected according to approved protocols. Mice were transcardially perfused with saline buffer, and the organs were removed and stored in 4 % formaldehyde for 2 days and then paraffin embedded. Four-micrometer sections of FtMt^+/+^ and FtMt^−/−^ mouse testes were deparaffinized in xylene and rehydrated through a series of alcohol gradients. Sections underwent antigen retrieval with 0.05 % protease type XIV digestion for 5 min at 37 °C, and endogenous peroxidase activity was quenched by 0.3 % H_2_O_2_ in methanol for 20 min. The tissues were incubated for 1 h at room temperature with rabbit anti-mouse FtMt antiserum [16] diluted 1:1,000 and then with Rabbit-on-Rodent HRP-Polymer (BIOCARE Medical, CA, USA) for 30 min. Sections were then incubated for 5 min with 3,3′-diaminobenzidine (DAB), washed, counterstained with hematoxylin, dehydrated, and coverslipped.

### Light and electron microscopy

Small blocks of heart were fixed in 3 % glutaraldehyde (Acros Organics, Thermo Fisher Scientific, Waltham, MA, USA) in Sorensen phosphate buffer (0.1 M; pH 7.4) overnight at 4 °C, post-fixed with 1 % osmium tetroxide in 0.1 M Sorensen phosphate buffer for 30 min, dehydrated, and embedded in Araldite (Fluka-Sigma Aldrich). Semi-thin sections, 2-μm thick, were stained with toluidine blue. For electron microscopy, ultrathin sections (200 nm) were obtained with an Ultracut ultramicrotome (Reichert-Jung, Leica, Microsystems GmbH, Wetzlar, Germany), stained with uranyl acetate and lead citrate, and observed with a JEM 1010 transmission electron microscope (Jeol, Tokyo, Japan).

### TBARS assay

Lipid peroxidation was assessed using the thiobarbituric acid reactive substance (TBARS) assay. Heart tissue was homogenized in 150 mM KCl, and an aliquot was used for the determination of protein concentration. A 100-μl aliquot of tissue homogenate in triplicate was mixed with 0.5 ml of 0.22 M butylated hydroxytoluene (Sigma), 3 ml of 1 % phosphoric acid, and 1 ml of 0.6 % thiobarbituric acid (Sigma). Samples were incubated at 100 °C for 60 min and then cooled at room temperature. Lipids were extracted using 5 ml of isopropanol:chloroform (11:7, *v*/*v*), centrifuged at 2,000×*g* for 10 min, and the absorbance of the upper layer was read at 535 nm. The amount of TBARS was quantified using a standard curve of malonaldehyde bis (dimethyl acetal), (MDA, Sigma).

### Caspase activity assay

Caspase-3 activity was determined using the ApoTarget Caspase Colorimetric Assay kit (Invitrogen, Monza, Italy), following the manufacturer’s protocol. In brief, tissue samples were lysed in 100 μl of lysis buffer and protein concentrations in samples determined using the Bio-Rad protein assay. After incubation on ice for 10 min, the samples were centrifuged at 16.000×*g* for 3 min at 4 °C. Each supernatant was mixed with 50 μl of 2X reaction buffer/DTT mix and 5 μl of 1 mM caspase-3 substrate (DEVD-pNA, 50 μM final concentration), and then, the samples were incubated for 2 h at 37 °C in the dark. Developed color was measured at 405 nm, and caspase activity was calculated in terms of absorbance units per microgram protein.

### Oxidized protein detection

Hearts were homogenized in lysis buffer (Tris HCl 20 mM, pH 7.4, 0.1 % SDS, and protease inhibitor). After centrifugation at 13.000 rpm for 10 min at 4 °C, the supernatant was added with 50 mM DTT, frozen, and stored at −20 °C. Oxidized proteins in protein extracts were detected using the OxyBlot Protein Oxidation Detection Kit (Millipore) following manufacturer’s instructions. In brief, the samples were reacted with 2,4-dinitrophenylhydrazine (DNPH) for derivatization to 2,4-dinitrophenylhydrazone (DNP), then were loaded on SDS-PAGE, blotted, and incubated with an anti-DNP antibody. The bound activity was revealed by ECL (GE Healthcare).

### Statistical analysis

Differences were analyzed using a Student’s *t* test for paired samples, and comparisons were made using appropriate analysis of variance (ANOVA). The significance level was set to *p* < 0.05.

## Results

### Production of the FtMt^−/−^ mice

**Fig. 1:**
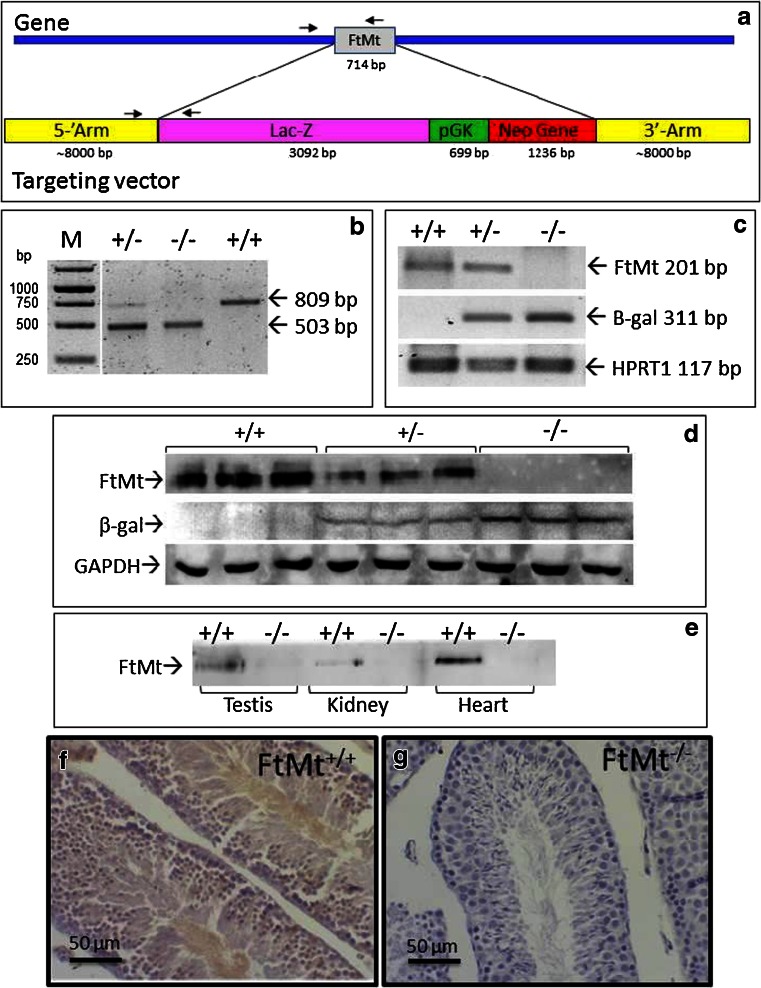
Construction and genotyping of FtMt^−/−^ mice. **a** Schematic of the FtMt locus and targeting vector, the *arrows* indicate the PCR primers for genotyping. **b** Genotyping gel of mice wild type (+/+), heterozygous (+/−), and homozygous (−/−) for the inactivated FtMt allele. In the M lane, a DNA molecular weight ladder was loaded. The size of the expected PCR amplicons is indicated. **c** RT-PCR analysis of testis RNA extracted from wild type (+/+), heterozygous (+/−), and homozygous (−/−) mice and amplified with primers for FtMt, beta-galactosidase (β-gal, encoded by the Lac-Z gene), and the housekeeping transcript HPRT1. **d** Immunoblotting of protein extracts from the testis of wild type (+/+), heterozygous (+/−), and homozygous (−/−) mice overlaid with antibodies for mouse FtMt, β-gal, and GAPDH as loading calibrator. **e** Immunoblotting of protein extracts from various tissues of wild type (+/+) and homozygous (−/−) mice, overlaid with antibodies for mouse FtMt. (**f**,**g**) Immunohistochemical detection of FtMt in the testis of wild type (**f**) and FtMt(−/−) (**g**) mice. *Bar* 50 μm

The structure of the gene targeting construct is shown in Fig. 1a. The intronless FtMt gene of 714 bp was replaced by a 5,030-bp cassette that contained lac-Z gene under the control of the FtMt promoter and the gene for neomycin resistance under the control of the strong pGK promoter. Genotyping was done by PCR using a common Fwd primer and specific Rev primers for wild type (wt) or recombinant gene (Fig. 1). This allowed to distinguish the FtMt^+/+^, from FtMt^+/−^ and FtMt^−/−^ strains (Fig. 1b). To verify the absence of FtMt expression, we initially analyzed the testis, where its level is the highest [16]. RT-PCR analyses showed the absence of FtMt transcript and the presence of β-gal transcript encoded by the Lac-Z gene in the FtMt^−/−^ mice. The FtMt, but not the β-gal transcript, was present in FtMt^+/+^, while FtMt^+/−^ displayed both transcripts, as expected (Fig. 1c); possibly because of the low level of expression of FtMt, or of modification of the methylation status, we could not observe any β-gal activity in the testis of the FtMt^−/−^ mice or in any other tissue (results not shown). Western blotting of testis homogenates detected a FtMt band in the FtMt^+/+^ mice, but not in the FtMt^−/−^ mice, and a β-gal band in FtMt^−/−^, but not in FtMt^+/+^ mice (Fig. 1d). It confirmed also the absence of FtMt in the kidney and, more important, in the cardiac tissue of FtMt^−/−^ mice (Fig. 1e). As a further control, we performed immunohistochemical staining of slices from the testis. A stain was evident in the seminiferous tubule of the FtMt^+/+^ mice that corresponded to the spermatocytes, whereas no staining was observed in the testis of FtMt^−/−^ mice. (Fig. 1f, g). This also confirmed the specificity of the antibody.

### FtMt^−/−^ phenotype

**Fig. 2:**
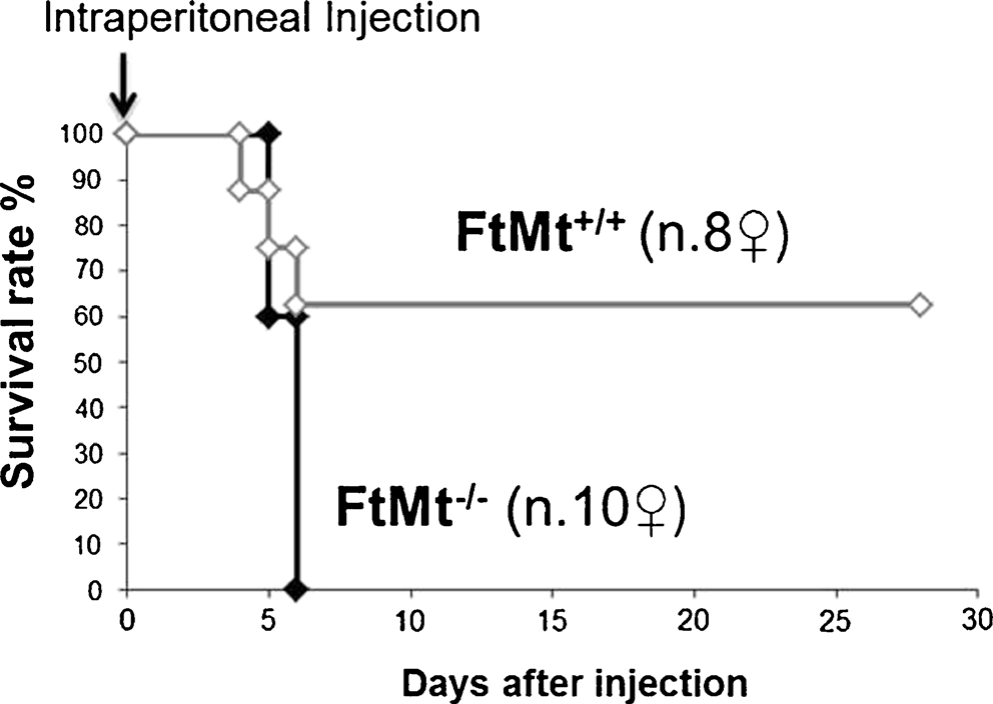
Cumulative survival in mice treated with Dox. Kaplan-Meier plot of animal survival. Twelve-week-old female mice (8 FtMt^+/+^ and 10 FtMt^−/−^) were subjected to a single intraperitoneal injection of 15 mg/kg Dox

The FtM^**+/−**^ and FtMt^−/−^ mice did not show any evident phenotype. They consumed the expected amount of food and behaved normally, also after 16 months of age. We did not observe any overt defect in fertility, both in males and females FtMt^−/−^. This corresponds to the description of FtMt^−/−^ mice in the C57B/6J strain [22]. FtMt is known to be preferentially expressed in the testis, heart, kidney, and some neurons [16]. Being FtMt highly expressed in the heart, we suspected that the heart of FtMt-deficient animals may be more sensitive to cardiotoxic drugs. To test this hypothesis, we treated the mice with Dox, which is a well-known cardiotoxic drug [23–25]. In our experimental protocol, we used the same dose and time-point recently used by us to investigate acute Dox cardiotoxicity [36] and we treated 12-week-old (84-day) female mice with a single intraperitoneal injection of 15 mg/kg to induce acute toxicity. The Kaplan-Meier survival plot reported in Fig. 2 shows that upon Dox challenge, the mortality of FtMt^−/−^ mice was much higher than that of wild type animals. None of the mice without FtMt survived more than 6 days, whereas one third of FtMt^+/+^ animals was still alive more than 3 weeks after treatment.

**Fig. 3:**
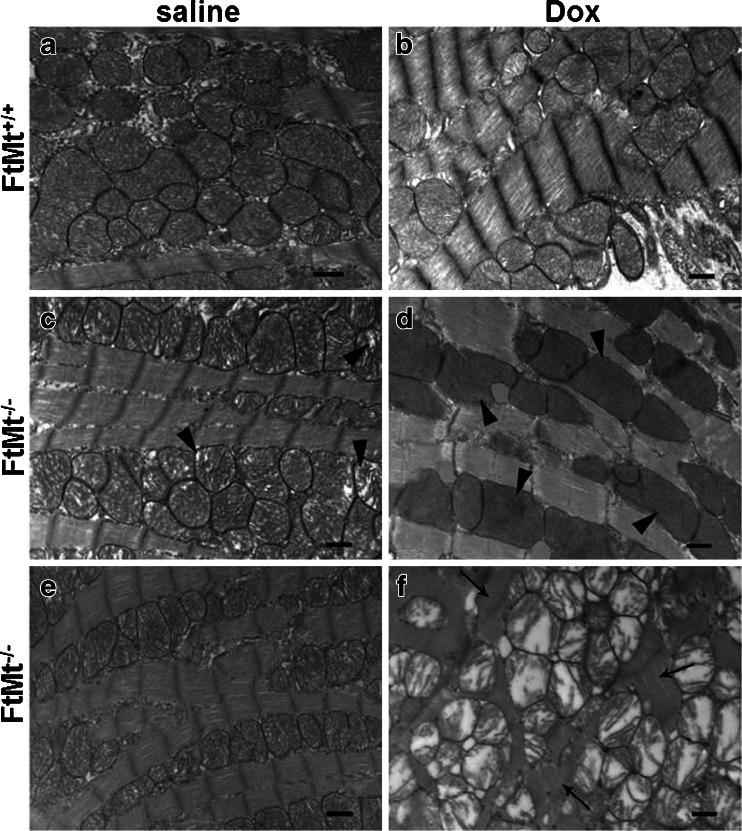
Heart morphological evaluation in mice treated with Dox. Transmission electron microphotographs of hearts from wild type mice treated with saline (**a**) or Dox (**b**), and FtMt^−/−^ mice treated with saline (**c** and **e**) or Dox (**d** and **f**). *Arrowheads* indicate damage/absence of cristae; *arrows* indicate fibril disarrangement. *Bars* 500 nm. Three animals for each group and three sections for each sample were examined, and representative images are shown

To further evaluate the role of FtMt in Dox-dependent cardiotoxicity, wild type and FtMt^−/−^ female mice were treated with saline or Dox (15 mg/kg) and sacrificed 4 days later; no mortality was observed, in line with the findings of our previous study [36]. Transmission electron microscopy analysis did not show evident differences between the hearts of control wild-type mice before and after Dox treatment (Fig. 3a, b). Both the fibril organization and the mitochondrial morphological features were comparable in these two groups. Also, the histopathogical hallmarks of anthracycline cardiotoxicity were absent in the treated FtMt^+/+^ group [37]. In contrast, the hearts of FtMt^−/−^ mice showed some defects: in the untreated ones, scattered mitochondria had incomplete cristae (Fig. 3c, arrowheads) although fibrils were regularly arranged (Fig. 3e). After Dox treatment, the damage was more severe with condensation and fragmentation of most myofibrils in some fields (arrows in Fig. 3f). Also, mitochondrial damage was more evident with zones in which the cristae were absent (Fig. 3d, arrowheads) and others with cristae completely disrupted (Fig. 3f). This type of morphology was observed in all the three animals analyzed in TEM. In no sample we could detect evident morphological signs suggesting apoptosis, e.g., chromatin condensation, nuclear fragmentation, and apoptotic bodies. Observation at the light microscopic level of toluidine blue-stained heart semi-thin sections of FtMt^+/+^ mice exposed to Dox or saline, and untreated FtMt^−/−^ mice did not show any significant alteration, and the morphology resulted in line with the normal myocardial structure. Similarly, FtMt^+/+^ and untreated FtMt^−/−^ mice showed a regular myofibrils appearance (results not shown). Only in FtMt^−/−^ mice fibril disorganization was evident, in line with the results of electron microscopy in Fig. 3f (results not shown).

### FtMt^−/−^ biochemical analyses

**Fig. 4:**
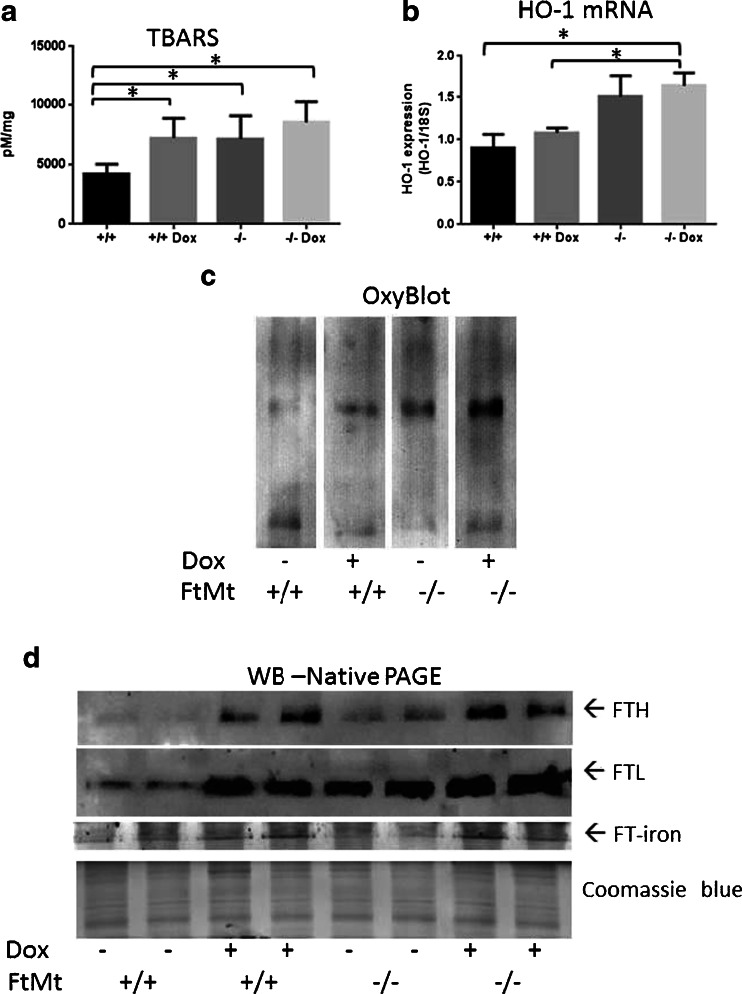
Indices of oxidative stress and iron status in the heart of Dox-treated mice. Heart extracts from 8-week-old wild-type (+/+) and FtMt-deficient mice (−/−) treated with Dox 15 mg/kg or saline and sacrificed after 4 days were analyzed. Oxidative stress was evaluated by measuring cardiac lipid peroxidation with the TBARS assay (**a**), HO-1 mRNA expression by qRT-PCR (**b**) and oxidized proteins by oxyblot (**c**). Ferritin was detected by Western blotting of non-denaturing PAGE using specific antibodies for H (FTH) and L ferritin (FTL); for evaluation of ferritin iron, the gels were stained with Prussian blue and then the color enhanced using DAB and H_2_O_2_ (Ft-iron) (**d**). Coomassie blue stain of the gels is shown for load calibration. At least five animals per group were analyzed, two representative samples are shown

Since ROS formation plays a role in Dox cardiotoxicity [23, 25, 37], we measured markers of oxidative stress in the hearts of the mice. TBARS, index of lipid peroxidation and oxidative damage, was significantly increased by Dox treatment in FtMt^+/+^ mice, as expected, and it was also significantly higher in untreated FtMt^−/−^ mice, in which it was further increased, but not to a significant level, by Dox treatment (Fig. 4a). Also, heme oxygenase-1 mRNA (HO-1), which is a marker of oxidative stress [38], was upregulated of about 2-fold in FtMt^−/−^ mice, and Dox induced a small, not significant increase in HO-1 mRNA levels in both wild-type and FtMt^−/−^ mice (Fig. 4b). The level of oxidized proteins, as assessed by OxyBlot, was low in the hearts of wild-type animals and increased progressively with Dox and loss of FtMt, with a maximum in the FtMt^−/−^ mice exposed to Dox (Fig. 4c). We analyzed also the ferritins, since they are upregulated by Dox in H9c2 cardiomyocytes and mouse heart [36, 39]. Both H and L ferritins increased after exposure to Dox, as expected, more interesting, the basal level of both ferritins was higher in FtMt^−/−^ than in FtMt^+/+^ (Fig. 4d). Ferritin iron, determined by enhanced Prussian blue stain of non-denaturing PAGE, mirrored the level of ferritins (Fig. 4d).

**Fig. 5:**
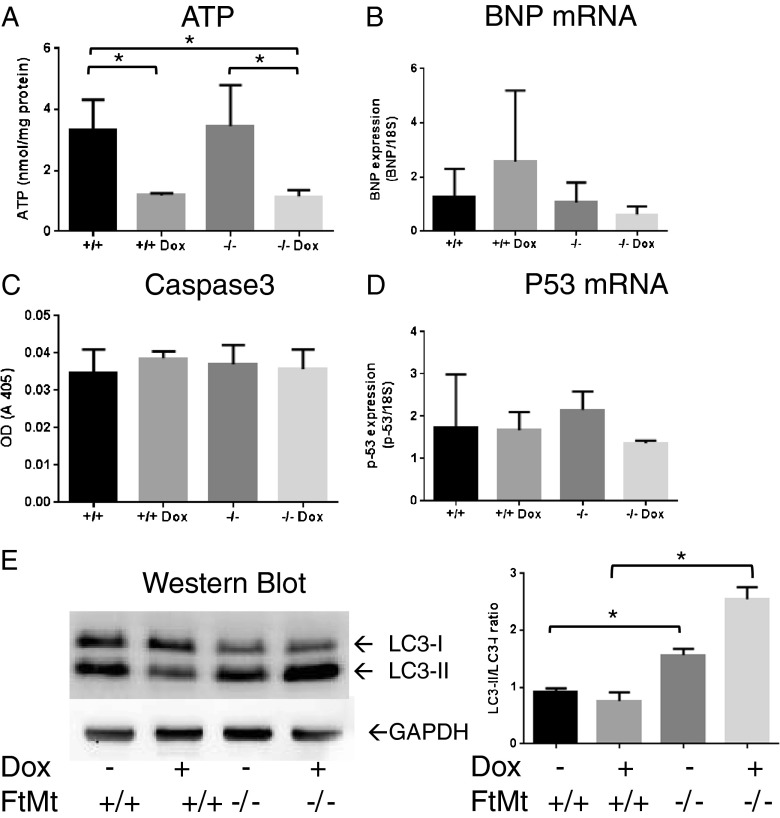
Indices of functionality, apoptosis, and autophagy in the heart of Dox-treated mice. Mitochondrial function and tissue damage were evaluated by measuring ATP content (**a**) and BNP mRNA expression (**b**), respectively. Apoptosis was evaluated by measuring caspase-3 activity (**c**) and p53 mRNA expression (**d**). For RT-PCR analysis of mRNA levels, samples were analyzed in triplicate and normalized to the housekeeping gene 18S RNA. Autophagy was evaluated by analyzing LC3 (**e**): the native (LC3-I) and lipidated (LC3-II) bands were separated on SDS-PAGE and revealed by immunoblotting with anti-LC3 antibody, GAPDH was used for load calibration. The graph shows the ratio between the intensity of the lower and the upper band calculated from three independent experiments

The mitochondrial damage indicated by electron microscopy observations prompted us to evaluate ATP level as an indicator of mitochondrial functionality (Fig. 5a). Treatment with Dox resulted in a significant decrease in ATP levels compared with the controls both in wild-type and FtMt-deficient mice. No difference was found in basal levels of ATP in the two mouse lines. We also analyzed the expression of B-type natriuretic peptide (BNP), a marker of cardiac injury [36, 40], and we found that BNP mRNA levels were similar in both wild-type and FtMt^−/−^ mice, and were changed by Dox challenge in opposite way in the two strains, but the difference was not significant (Fig. 5b). Given that DOX is a well-known inducer of apoptosis [24], we investigated whether apoptosis was preferentially induced in FtMt^−/−^ mice; however, in FtMt-deficient mice, caspase-3 activity was not different from that of controls and was not significantly affected by Dox treatment (Fig. 5c); moreover, p53 mRNA levels were similar in the four groups of animals (Fig. 5d). These results are in line with the lack of morphological features of apoptosis (e.g., chromatin condensation) indicated by EM (see Fig. 3).

Autophagy is a cellular process that removes damaged structures and thus provides a survival advantage to cells experiencing stress or nutrient deprivation, but excessive autophagy may cause cell death; thus, careful regulation of autophagy is important, particularly in the myocardium. Therefore, we investigated autophagic signaling by analyzing LC3, a marker of autophagosome formation that has been recently found to be induced in the skeletal muscle of rats 24 h after treatment with 15 mg/kg Dox [41]. Blotting of the heart extracts showed a proportional increase of the lipidated LC3-II band in the FtMt^−/−^ animals (50 % increase in LC3-II/LC3-I ratio), compared with the FtMt^+/+^ that was further stimulated by Dox up to >2-fold increase (Fig. 5e) suggesting a higher autophagy activity. Dox did not affect LC3 in the FtMt^+/+^ mice.

Dox is known to be toxic also to testis [42]; therefore, we performed an initial morphological analysis of the testis of 8-week-old mice sacrificed 30 days after Dox treatment. Histological examination of semi-thin slices showed that the testis of Dox-treated FtMt^+/+^ had a normal morphology with tubules rich in spermatocytes, similar to those shown in Fig. 1f or g, while the morphology of testis from Dox-treated FtMt^−/−^ mice was largely altered with complete absence of spermatocytes (Fig. S1).

## Discussion

FtMt occupies a strategic position in a site where the encounter between Fe(II) and ROS is very likely to occur and Fenton reaction to develop [43]. FtMt with its capacity to remove both Fe(II) and H_2_O_2_ is expected to protect the mitochondria from the development of toxic-free radicals. This hypothesis was confirmed by in vitro studies of cells that have been transfected to express FtMt at rather high levels [8]. However, FtMt is expressed only in mammals, in Drosophila [44] and also in plants [45], while it is absent in most vertebrates. Moreover, in mammals, it is expressed only by a few cell types, typically characterized by high oxidative activity. The finding that FtMt-deficient mice [22] and also FtMt-deficient Drosophila [44] are healthy and do not show any evident phenotype suggested that the functionality of FtMt is not essential under normal conditions. Moreover, a study on the DNA variations of human FtMt in patients affected by myelodysplastic syndromes and by movement disorders identified some variations, but no disabling mutations [46]. To verify the role of this gene, we produced a novel strain of FtMt-null mice, using a strategy slightly different from that used previously [22]. We used Lac-Z as a reporter gene, but we could not observe any B-gal activity in the testis of the FtMt^−/−^ mice or in any other tissue, possibly because of the low level of expression of FtMt, or to modification of the methylation status. Our mice are healthy and iron homeostasis appears to be preserved, as described before in a different FtMt^−/−^ strain [22].

We hypothesized that FtMt may be required under stress conditions by tissues expressing it. Heart is rich in FtMt, and FtMt protects HeLa cells from Dox [33], a well-characterized cardiotoxic agent. Moreover, Dox induces FtMt expression in cultured rat cardiomyocytes and causes mitochondrial iron accumulation [34]. To challenge the protective activity of FtMt in vivo in the heart, we tested a condition of acute cardiotoxicity by treating the mice with a dose of 15 mg/kg of Dox, as before [36]. Dox toxicity is strongly related to the age and gender of the mice. Thus, we restricted to 12-week-old (84 days) female mice and found that under these conditions the treatment killed all the FtMt^−/−^ mice, but only about half of the control animals, suggesting that FtMt is protective against Dox-mediated injury.

Histological examination under light (not shown) and electron microscopy (Fig. 3a, b) did not reveal morphologic abnormalities in hearts of the Dox-treated FtMt^+/+^ mice. However, about half of these mice were expected to die in the week after the treatment (Fig. 2). The lack of abnormalities is in agreement with published data that cardiac morphological alterations were evident after higher Dox dosages [47, 48], longer treatments [49], or repeated doses [50]. On the other hand, the FtMt^−/−^ hearts showed mitochondrial defects even when untreated, with loss of cristae in a part of mitochondria, but the mice survived without evident health problem until 18 month of age. More important, the Dox treatment aggravated the heart abnormalities causing major alterations of mitochondrial cristae and myofibril damages in some heart portions (Fig. 3c, d). Unfortunately we could not perform direct studies on heart functionality, and thus, we could not verify experimentally that cardiac failure was the cause of the mortality of the three control mice and of the ten FtMt-deficient mice described in Fig. 2. We are aware that this is a major limitation to this study. However, we thought reasonable to infer that mice exposed to toxic doses of a cardiotoxic drug would die for heart problems. Therefore, we performed biochemical analyses on the hearts. They revealed that FtMt^−/−^ hearts have increased lipid peroxidation, protein oxidation, and HO-1 expression, all indices of oxidative damages. We expected that the morphological alteration of the mitochondria in the FtMt-null mice was associated with a reduced functionality, but the level of ATP was very similar in the two strains. Also, the expression of cardiac BNP, an index of heart failure, was unchanged in the two strains, Moreover, we did not detect differences in indices of apoptosis, caspase-3, and p53 mRNA in the two hearts. The differences involved markers of iron status (cytosolic ferritins) and an important index of autophagy, LC3 [51]. Both increased in the FtMt-null mice. FtMt has a strong iron withdrawing capacity; thus, its absence may increase cytosolic iron and induce H and L ferritin expression, as it occurs in cultured cells [7], and also reduce the stabilization of HIF-1 and the expression of protective genes [52]. Apparently, the increase of the antioxidant activity of H-ferritin is not sufficient to compensate the loss of that associated with FtMt. Probably more interesting was the increase of lipidated LC3. Recently, it was shown that iron chelation is a strong inducer of mitophagy in cells [53], and possibly, the local iron deregulation caused by FtMt absence might favor this process. Altogether, the data indicate that the absence of FtMt affects mitochondrial morphology, increases oxidative damage and possibly autophagy, and has an effect on cytosolic ferritins.

Dox treatment in the control mice increased oxidative damage (TBARS and Oxyblot), reduced ATP, and induced cytosolic ferritin expression, in agreement with previous studies [36]. Surprisingly, the absence of FtMt did not have a significant effect on the response to the treatment, except for higher increases in protein oxidation and LC3 induction. We found that both testes and hearts were sensitive to the Dox treatments (supplemental data), suggesting a role for FtMt in the protection against the doxorubicin-induced mitochondrial iron accumulation that was indicated responsible for cardiotoxicity [34]. In fact, mitochondrial iron accumulation occurs also in cellular models of Freidreich’s ataxia where FtMt expression was protective [9, 10].

In conclusion, the present data indicate that the absence of FtMt alters the morphology of heart mitochondria with signs of oxidative damage. These mitochondria are more sensitive to Dox-induced damage, which results in a dramatic reduction of the mice survival, possibly due to heart failure. Notably, under our experimental conditions, heart damage 4 days after exposure to Dox is limited in wild-type mice, with no apoptosis and normal cardiac morphology. This suggests that FtMt may play a protective role under a number of not particularly severe pathophysiological conditions commonly experienced by the heart. Finally, the results of this study represent the first in vivo evidence for a function of FtMt.

## Electronic supplementary material

Below is the link to the electronic supplementary material.

ESM 1 (PDF 129 kb)
