# Supplementary material for: Mice lacking mitochondrial ferritin are more sensitive to doxorubicin-mediated cardiotoxicity
Source: J Mol Med (Berl). 2014 Apr 13;92(8):859–69. doi: 10.1007/s00109-014-1147-0 (PMC4118045; doi:10.1007/s00109-014-1147-0)
Supplement: Supplementary file 1 — (PDF 129 kb) [file 109_2014_1147_MOESM1_ESM.pdf]

J Mol Med 2014, Mice lacking mitochondrial ferritin are more sensitive to Doxorubicin-mediated cardiotoxicity, By Maccarinelli F, Gammella E, Asperti M, Regoni M, Biasiotto G, Turco E, Altruda F, Lonardi S, Cornaghi L, Donetti E, Recalcati S, Poli M, Finazzi D, Arosio P, Cairo G.

Supplementary

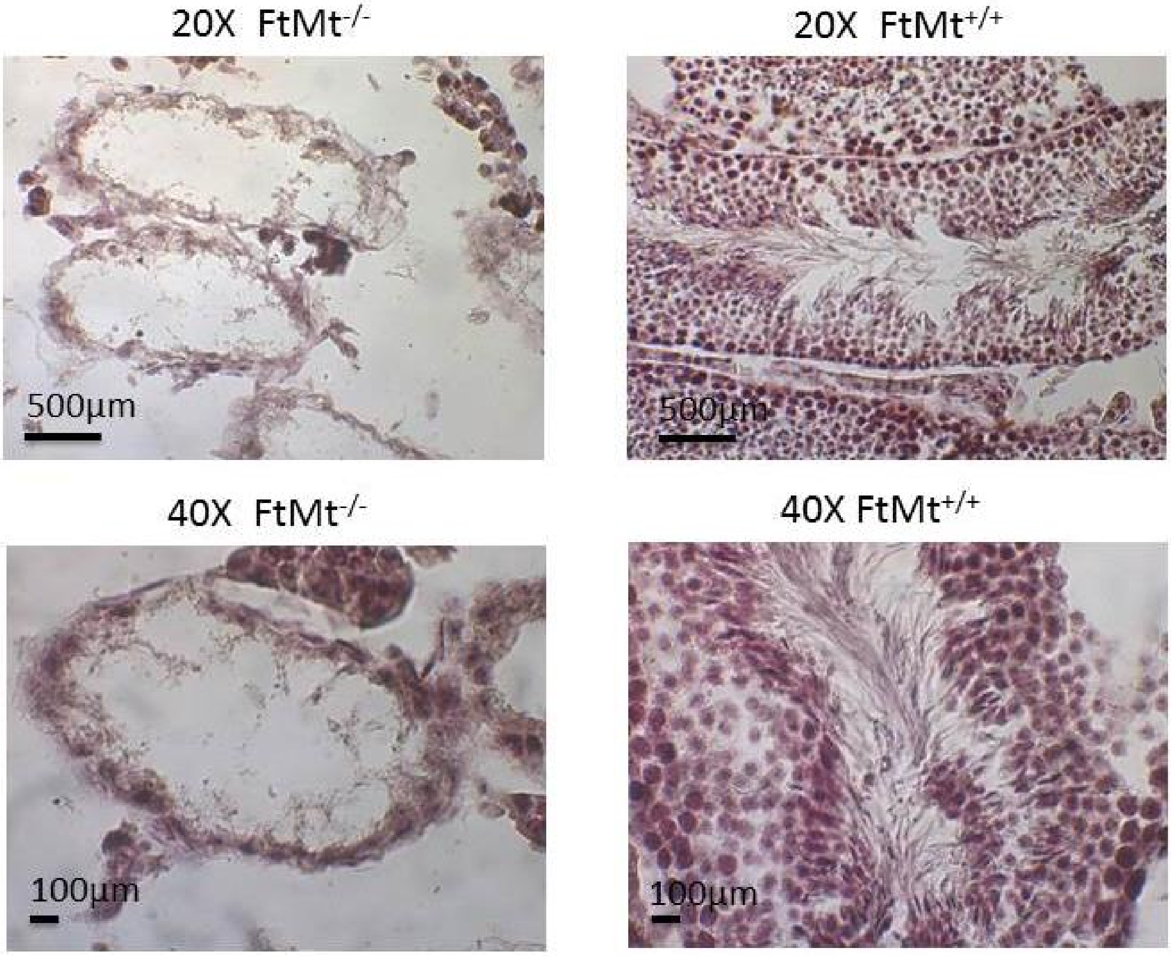

Fig. S1. The testis of FtMt$^{-/-}$ mice are more sensitive to Doxorubicin than those of wild type mice. Histology of the testis of mice sacrificed 30 days after treatment with 15 mg/kg Doxorubicin. The morphology of FtMt$^{+/+}$ mice appears unaffected by the treatment, while in the FtMt$^{-/-}$ spermatocytes are almost completely absent.

Fig. S1
